# Metabolic labeling of enterovirus 71 with quantum dots for the study of virus receptor usage

**DOI:** 10.1186/s12951-021-01046-5

**Published:** 2021-09-28

**Authors:** Xianliang Ke, Chunjie Li, Dan Luo, Ting Wang, Yan Liu, Zhongyuan Tan, Mingyuan Du, Zhike He, Hanzhong Wang, Zhenhua Zheng, Yuan Zhang

**Affiliations:** 1 CAS Key Laboratory of Special Pathogens and Biosafety, Center for Biosafety Mega-Science, Wuhan Institute of Virology, Chinese Academy of Sciences, Wuhan, 430071 China; 2 Department of Gastroenterology, Wuhan Children’s Hospital, Tongji Medical College, Huazhong University of Science and Technology, 430015 Wuhan, China; 3 The Center for Biomedical Research, Tongji Hospital, Tongji Medical College, Huazhong University of Sciences and Technology, Wuhan, 430100 China; 4 College of Chemistry and Molecular Sciences, Wuhan University, 430072 Wuhan, China

**Keywords:** EV71, Quantum dots, Nanobiotechnology, Metabolic labeling, ANL, Heparan sulfate, SCARB2

## Abstract

Fluorescent labeling and dynamic tracking is a powerful tool for exploring virus infection mechanisms. However, for small-sized viruses, virus tracking studies are usually hindered by a lack of appropriate labeling methods that do not dampen virus yield or infectivity. Here, we report a universal strategy for labeling viruses with chemical dyes and Quantum dots (QDs). Enterovirus 71 (EV71) was produced in a cell line that stably expresses a mutant methionyl-tRNA synthetase (MetRS), which can charge azidonorleucine (ANL) to the methionine sites of viral proteins during translation. Then, the ANL-containing virus was easily labeled with DBCO-AF647 and DBCO-QDs. The labeled virus shows sufficient yield and no obvious decrease in infectivity and can be used for imaging the virus entry process. Using the labeled EV71, different functions of scavenger receptor class B, member 2 (SCARB2), and heparan sulfate (HS) in EV71 infection were comparatively studied. The cell entry process of a strong HS-binding EV71 strain was investigated by real-time dynamic visualization of EV71-QDs in living cells. Taken together, our study described a universal biocompatible virus labeling method, visualized the dynamic viral entry process, and reported details of the receptor usage of EV71.

**Supplementary Information:**

The online version contains supplementary material available at 10.1186/s12951-021-01046-5.

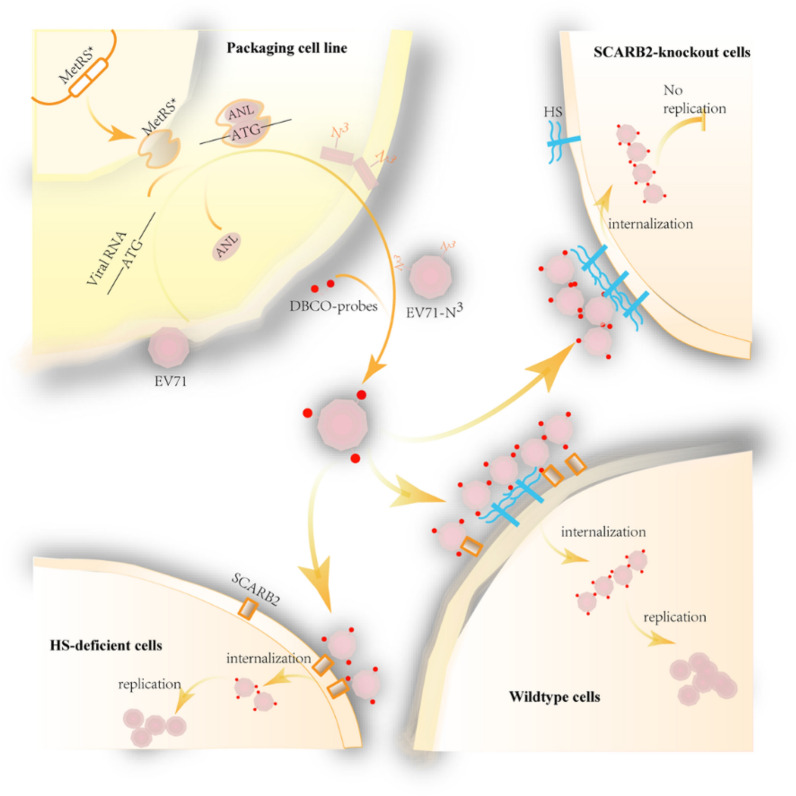

## Introduction

Human enterovirus 71 (EV71) is the major causative pathogen of the human hand, foot, and mouth disease (HFMD) [1]. EV71 infects millions of children in the Asian-Pacific area and causes thousands of deaths every year. Because HFMD often leads to severe neurological complications, such as brainstem encephalitis, meningitis, and poliomyelitis-like paralysis, EV71 is recognized as one of the most important neurotropic picornaviruses [1].

Currently, no effective drugs have been developed for the EV71 virus. The cell entry process is a key step for viruses to establish successful infection in cells and usually provides promising targets for antiviral therapeutic intervention [2]. In recent decades, many cellular molecules, such as vimentin, SCARB2, PSGL-1, nucleolin, fibronectin, and heparan sulfate proteoglycans, have been proven to serve as cellular receptors for EV71 infection [3–7]. However, the mechanism of cell entry mediated by the receptors, as well as viral preferences among them, are not fully understood.

Virus tracking in living cells provides powerful tools for the investigation of the cell entry mechanism of many viruses [8]. The most challenging step of virus tracking is labeling viral particles without affecting their replication and infectivity. By genetic fusion of fluorescent proteins (FPs) or conjugation of fluorescent probes to viral structural proteins, many viruses have been successfully labeled [9]. However, small-sized viruses such as EV71 have benefited little from this technique due to their virological characteristics. For example, the relatively small size of the EV71 particles (~ 28 nm) [1] and its structural proteins make FP-fusion an impossible mission. The molecular weight of the commonly used FPs (~ 27 kDa) is equal to or even exceeding that of the targeted viral proteins. So, fusion with FPs may easily prevent viral capsid protein monomers from multimerization into highly ordered viral particles. Consequently, this labeling strategy would be lethal for some small-sized viruses including EV71, thus, limited its application. As for chemical labeling, the target charged residues on viral particles are often essential for the receptor binding of EV71 [10, 11]. Several positively charged residues were reported to be responsible for virus binding to HS and PSGL-1 and even for virulence in patients and animal models [11–13]. Moreover, chemical labeling may interfere with the interaction between viruses and cells. To date, only a few studies focused on dynamic visualization of the EV71 infection process have been reported [14, 15].

Metabolic incorporation of unnatural amino acids (UAAs) is a novel strategy that provides opportunities to label viruses with minimal effects on virus infectivity. The methionine (Met) analog azidohomoalanine (AHA) is the most commonly used UAA for virus labeling. AHA can bind to the endogenous methionyl-tRNA synthetase (MetRS) instead of Met [16], thus, can be incorporated into viruses during viral protein translation. The progeny virus can be labeled with fluorescent probes through reaction with the azido group [14, 17–19]. However, the affinity of AHA to MetRS is 390-500-fold lower than that of Met. To eliminate the low labeling efficiency caused by the competition between AHA and Met, AHA-base virus labeling must be performed in Met-free medium [17, 19], which induces substantial protein abundance differences [20] and may affect cell growth or even virus yield significantly [21]. UAAs can also be incorporated into virus proteins through genetic code expansion in mammalian cells. However, the labeling system requires not only engineered aaRS/tRNA expression cassettes but also eRF1 mutants to improve the incorporation efficiency [22], making the whole system rather complicated.

Long-term dynamic tracking of the labeled virus is often limited by the photobleaching of traditional chemical dyes or FPs. Quantum dot (QD), an inorganic nanoparticle, is an alternative fluorescent material suitable for long-term imaging owing to its remarkable brightness and photostability. It has been widely used for the dynamic tracking of many viruses including retrovirus [23] and influenza virus [24].

**Scheme 1:**
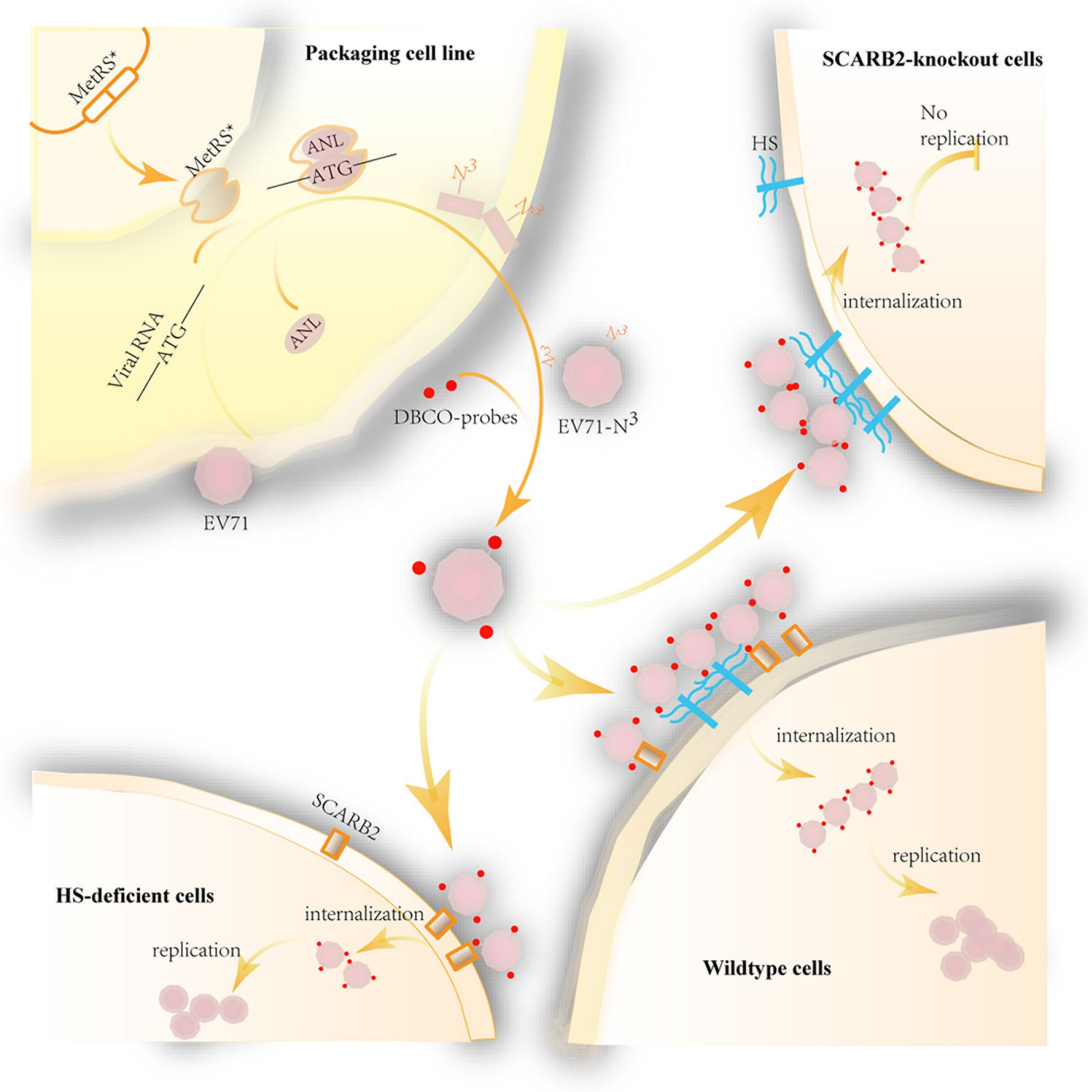
Bioorthogonal labeling of EV71 to investigate the roles of different receptors in infection. RD cells were engineered to express MetRS*, which translated the ATG codon on viral RNA to ANL in viral proteins. Thus, the progeny virus bearing azido groups (EV71-N3) can be labeled with DBCO probes. The labeled virus was then used to infect wild-type or receptor-knockout cells to comparatively study the function of the receptors. In the next infection cycle, the entry dynamics of the labeled viruses could be observed directly by microscopy

In this study, we developed a novel virus labeling method and accomplished long-time dynamic visualization of the cell entry of EV71 through QD-labeling (Scheme 1). The labeling technique relies on a mutant MetRS and azidonorleucine (ANL) [25], another Met surrogate, to label viral particles during virus production. MetRS with an amino-acid binding site mutation (MetRS L274G) enables the Met tRNA to be charged with ANL instead of Met efficiently without the requirement of Met deletion [25]. When cells that express MetRS L274G were applied for virus production, cellular proteins, as well as the progeny viral proteins, could be modified by incorporating ANL. Then ANL-carrying viruses can be readily labeled with dibenzocyclooctynol (DBCO) probes including DBCO-QDs through click chemistry under physiological conditions with high efficiency [26]. Using the labeled viruses, the functions of two EV71 receptors during infection, SCARB2, and heparan sulfate (HS) were comparatively studied. Entry of a strong-HS-binding EV71 strain into SCARB2-knockout cells was also studied by long-term dynamic visualization.

## Methods

### Cell lines and viruses

RD (human rhabdomyosarcoma cells) and HeLa cell lines were maintained in Dulbecco’s modified Eagle’s medium (DMEM) supplemented with 10% fetal bovine serum (FBS). Vero (African green monkey kidney cells) cell line was maintained in minimum essential medium (MEM) supplemented with 10% FBS. Two SCARB2-knockout mouse fibroblast cell lines, namely, the L929-SCARB2KO and L929-EXT1KO cell lines, which were heparan sulfate deficient, were constructed as described previously [27]. L929, L929-SCARB2KO, and L929-EXT1KO cells were maintained in Dulbecco’s modified Eagle’s medium (DMEM) supplemented with 10% FBS. EV71 virus strain EV71 GZCII-P30 [27] was propagated in Vero or RD cells. After titering in RD cells, virus stocks were stored at -80 °C until use. 

### RD-MetRS* cell line construction

All molecular cloning work was performed in *E. coli* strain DH5α. Briefly, the human methionyl-tRNA synthetase (MetRS) coding region was amplified from the cDNA of HEK-293T cells using primer MetRS-F and MetRS-R. Then, a leucine to glycine mutation at amino acid 272 was introduced into MetRS by overlapping-PCR. Two DNA fragments were amplified using primer MetRS*-F(EcoRI) + MetRS*-2R or MetRS*-2 F + MetRS*-R(NotI) respectively, and then fused together by PCR using primers MetRS*-F(EcoRI) + MetRS*-R(NotI). The MetRS mutant was cloned into the PiggyBac transposon vector (PB513B-1, System Biosciences, USA) between enzyme sites EcoRI and NotI through restriction cloning to generate PB-MetRS*. Primer sequences are listed in Additional file 1: Table S1.

The plasmid PB-MetRS* was transfected into the RD cell line along with Super PiggyBac Transposase (PB200PA-1, System Biosciences, USA) expression plasmid at a ratio of 2:1. The transposase will move the transposon which harbors the MetRS* and Pac (puromycin resistance gene) gene to the cell genome for stable expression. Transfected cells were screened with 4 µg/mL puromycin (Sangon Biotech, China) for 2 weeks to select MetRS*-expressing cells, which were designated RD-MetRS* cells and used for virus labeling.

### Virus labeling and purification

RD-MetRS* cells were cultured in normal medium for 24 h. Then, EV71 was added at a multiplicity of 0.01 for infection at 37 °C for 1 h. After incubation, cells were cultured with normal medium supplemented with 1000 µM ANL for labeling. When full cytopathic effects (CPEs) were observed (approximately 24 h post-infection), the virus in the cell supernatant after 3 freeze-thaw cycles were collected for further purification. Briefly, cell supernatants were centrifuged at 5,000 rpm for 10 min, followed by 10,000 rpm for 30 min to remove cell debris. The cleared viruses were subjected to ultra-speed centrifugation at 27,000 rpm for three hours in an SW28 rotor (Beckman, Palatine, IL USA). Sediments were dissolved in PBS buffer (150 mM, pH 7.4) and mixed with DBCO-AF647 (Jena Bioscience, Germany) or DBCO-QDs for labeling at room temperature for 2 h. Then, the labeled virus was purified by density gradient centrifugation (DGC). Briefly, the labeled virus was loaded onto a noncontinuous gradient consisting of 20%, 30%, 40%, and 50% Optiprep (Cosmo Bio, Carlsbad, CA, USA) and separated by centrifugation at 38,000 rpm at 4 °C for four hours in an SW41 rotor (Beckman counter). Aliquots of 1ml each were taken from the top to the bottom of the centrifugation tube for the detection of both fluorescent signals and viral antigens.

### Transmission electron microscopy

For TEM examination, carbon-coated copper grids were immersed in purified virus solution or QDs for 20 min. Redundant liquid on the grids was removed using filter papers, and the grids were negatively stained with 1% phosphotungstate (10 µL) for 10 s. After drying at room temperature overnight, grid samples were examined under a Hitachi H7000 electron microscope.

### Virus titering and growth curve assay

Viruses were titered in RD cells using the TCID50 method. Briefly, serial 10-fold diluted viruses were used to infect monolayer RD cells in 96-well plates. At 72 h post infection (poi), positive wells that showed CPE were counted, and virus titers were calculated using the Muench and Reed method [28]. For the growth curve assay, RD cells were infected with EV71 virus at an MOI of 0.01. Viruses were collected at the indicated time points and then titered as described above.

### Immunofluorescence assay

Cells were seeded in a 35 mm glass-bottom dish. After 24 h, cells were infected with EV71. At different time points poi, cells were fixed with 4% polyformaldehyde at room temperature for 15 min, followed by permeation for 10 min with PBS supplemented with 0.02 % Triton X-100. After that, the cells were sequentially blocked with blocking buffer (PBS containing 3% BSA and 0.3% normal goat serum) and incubated with EV71 specific antibody (Abcam, USA) and fluorescent secondary antibodies. Then, the cells were observed with an UltraVIEW VoX double-disc live-cell fluorescence confocal microscope (PerkinElmer, Co). In some cases, cells were incubated with Hoechst 33,258 (Beyotime Biotech, China) to stain the nucleus before imaging. The Image pro plus software (version 6.0) was used for line profile analysis and the Fiji Image J software (imagej.nih.gov/ij/) was used for quantification of the fluorescent signals.

### qRT-PCR quantification of virus RNA copy number

Viral RNA was extracted with a Viral RNA Kit (Omega Biotek, USA). To quantify viral RNA copies, a pair of VP1-specific primers and TaqMan probes were applied for one-step qRT-PCR using the SuperScript™ III One-Step RT-PCR System with Platinum™ Taq (Invitrogen, USA) on a BioRad CFX96 qPCR Machine. The sequences of primers and probes are described in [27].

### Drug inhibition assay

Cells cultured in 35 mm glass-bottom dishes (Nest cop, China) were treated with medium containing DMSO or 50 mM chlorpromazine. Cells were then washed and incubated with EV71-QDs at an MOI of > 50 at 4 °C for 30 min. Then, the cells were washed twice with ice-cold PBS, and fresh medium containing DMSO or 50 mM chlorpromazine was added. Dishes were kept on ice until observation under an Andor spinning disk confocal microscope. During live-cell imaging, cell culture dishes were kept in a chamber that supplied 5% CO_2_ at 37 °C. Trajectory and velocity analyses were performed with Fiji ImageJ using the Trackmate plugin.

## Results

### Construction and characterization of the RD-MetRS* cell line

**Fig. 1:**
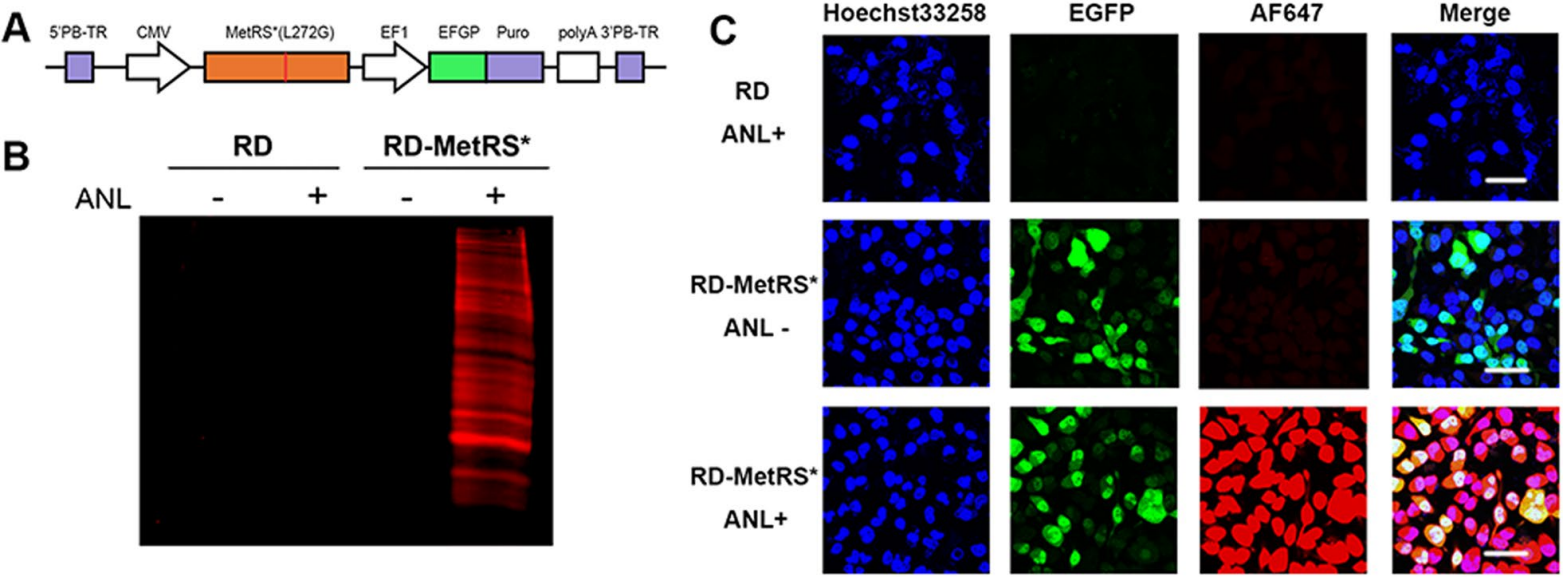
Construction and characterization of the MetRS mutant-based labeling system. **A** Scheme of the MetRS* expression plasmid PB-MetRS*. **B** Sodium dodecyl sulfate-polyacrylamide gel electrophoresis for the separation of cellular proteins of RD cells (lanes 1 and 2) and RD-MetRS* cells (lanes 3 and 4) cultured with or without ANL. The gel was stained with DBCO-AF647, and fluorescent signals were detected. **C** Wild-type RD cells and RD-MetRS* cells cultured with or without ANL were stained with DBCO-AF647 and observed under microscopy. Scale bar: 50 μm

An ANL-based virus labeling system was developed for the bioorthogonal labeling of viruses during infection (Scheme 1). We took advantage of the discovery that a mammalian MetRS mutant could charge methionine tRNA with ANL [25]. An expression cassette of the human MetRS mutant, termed MetRS* in this report, was inserted into the genome of RD cells to generate a cell line that could translate the ATG codon to ANL. When this cell line was used for virus production, ANL was incorporated into the viral structural protein, preparing the progeny virus for selective reaction with fluorescent molecules harboring a DBCO group through copper-free click chemistry. We first detected the ability of this cell line to incorporate ANL into cellular proteins. After integration of MetRS* into the cell genome by the PiggyBac transposon system (Fig. 1A), cells were cultured in an ANL-containing medium or normal medium as a control. Cell lysates were incubated with DBCO-AF647 dye, and the proteins were separated by sodium dodecyl sulfate-polyacrylamide gel electrophoresis. Strong AF647 signals could be detected in RD-MetRS* cells cultured in ANL medium but not in normal medium. In contrast, the cell lysate of wild-type RD cells could not be labeled with DBCO-647, regardless of whether ANL was present (Fig. 1B). ANL incorporation was also detected by staining the cells directly with DBCO-AF647. AF647 signals could be detected in nearly all ANL medium-cultured RD-MetRS* cells but not in wild-type RD cells or RD-MetRS* cells maintained without ANL (Fig. 1C). No background or nonspecific signals were detected in either the cell lysate or the labeled cells, indicating that wild-type MetRS in RD-MetRS*cannot use ANL for translation and that the MetRS*- ANL pairs are highly selective. Strong AF647 signals in ANL-cultured RD-MetRS* cells also suggested the high efficiency of both the incorporation of ANL during protein synthesis and posttranslational click labeling.

### The MetRS* labeling system does not require methionine depletion in the medium

Methionine in cell culture medium often competes with its functional surrogates in protein translation and reduces the efficiency of the incorporation of the UAAs as well as the labeling efficiency. When using AHA for biorthogonal labeling, cells are usually cultured in methionine-depleted medium and dialyzed FBS to enable efficient labeling [17, 19].

**Fig. 2:**
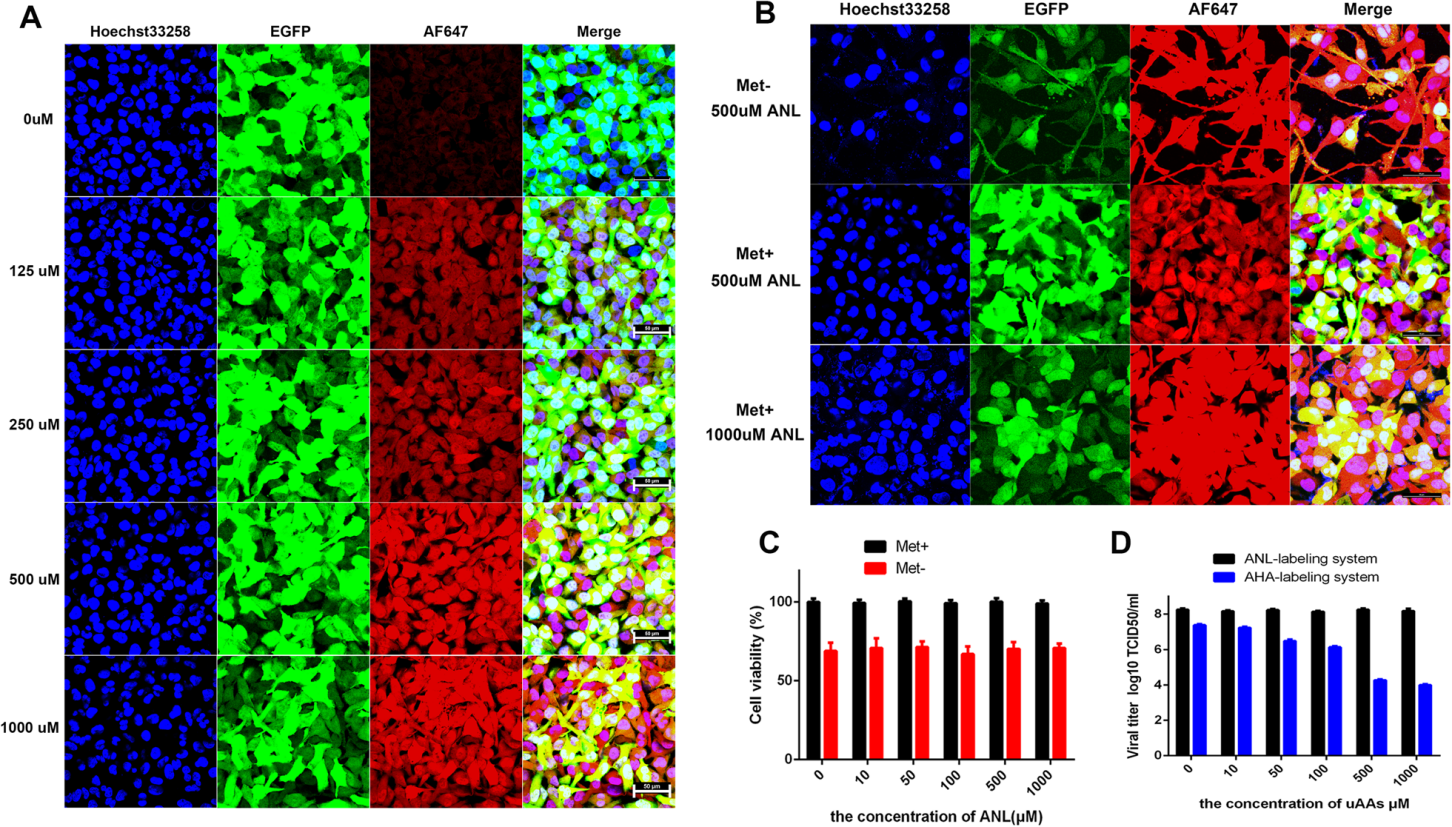
Effect of methionine depletion on protein labeling and virus production. **A** DBCO-AF647 labeling of RD-MetRS* cells cultured in normal medium supplemented with different concentrations of ANL. Fluorescent images of RD-MetRS* cells cultured in normal or Met- medium are shown in **B**. Scale bar: 50 μm. **C** The viability of RD-MetRS* cells cultured in normal (Met+) or methionine-free medium (Met-) supplemented with ANL at different concentrations was detected by CCK-8 assay, and the titers of viruses produced by AHA-based system (Met-AHA+) and ANL-based system (Met + ANL+) are shown in **D**

To prove that the cellular proteins can be labeled efficiently with the existence of Met through MetRS*-ANL labeling system, RD-MetRS* cells cultured in normal medium containing different concentrations of ANL were labeled with DBCO-AF647. AF647 signals gradually increased as the concentration of ANL increased from 0 to 1,000 µM (Fig. 2A), suggesting a concentration dependency of the labeling efficiency. When the concentration of ANL reaches 500 µM, the signal of AF647 was extremely strong.

Considering the possibility that wild-type MetRS may compete with MetRS* during protein translation and translate ATG to methionine, we also determined the necessity of using a Met-free medium for virus labeling. RD-MetRS* cells were cultured in a normal or methionine-free medium containing ANL. As expected, the AF647 signals in cells cultured with 500 µM ANL in the methionine-free medium were stronger than those in the normal medium group, but the cell morphology began to change when cultured with methionine-free medium for 1~2 days (Fig. 2B). However, when the concentration of ANL was increased to 1000 µM, the fluorescent signals in the normal medium group were comparable to those in the methionine-free group (Fig. 2B), proving that the effect of competitive usage of methionine by cells can be simply reversed by increasing the concentration of ANL. In addition, Cell Counting Kit-8 (CCK-8) assay showed a nearly 20% decline in cell viability when using ANL as a substitute for Methionine during prolonged incubation (Fig. 2C).

Moreover, we compared the titer of EV71-GZCII strain amplified by an AHA-based system, which requires a Met-depletion medium for labeling (Met-/AHA+), and our ANL-based system (Met+/ANL+) (Fig. 2D). In the absence of UAAs, the titer of viruses amplified by an ANL-based system is about 10 times higher than that of an AHA-based system due to no requirement of Met depletion, indicating methionine is essential for both cell growth and virus replication. When the concentration of AHA increased, the viral titer decreased significantly. Reduction of virus production by AHA in methionine-free medium was also observed during HSV labeling [21]. In contrast, no difference in cell viability (Fig. 2C) or virus yield (Fig. 2D) was observed when the ANL concentration was varied. This result is consistent with the result of the crystal violet staining assay (Additional file 1: Figure S1), indicating our ANL-based labeling system provides not only robust labeling efficiency but also high virus yield compared to other metabolic labeling strategies. And normal medium containing 1000 µM ANL was used for virus labeling in the RD-MetRS* cell line.

**Fig. 3:**
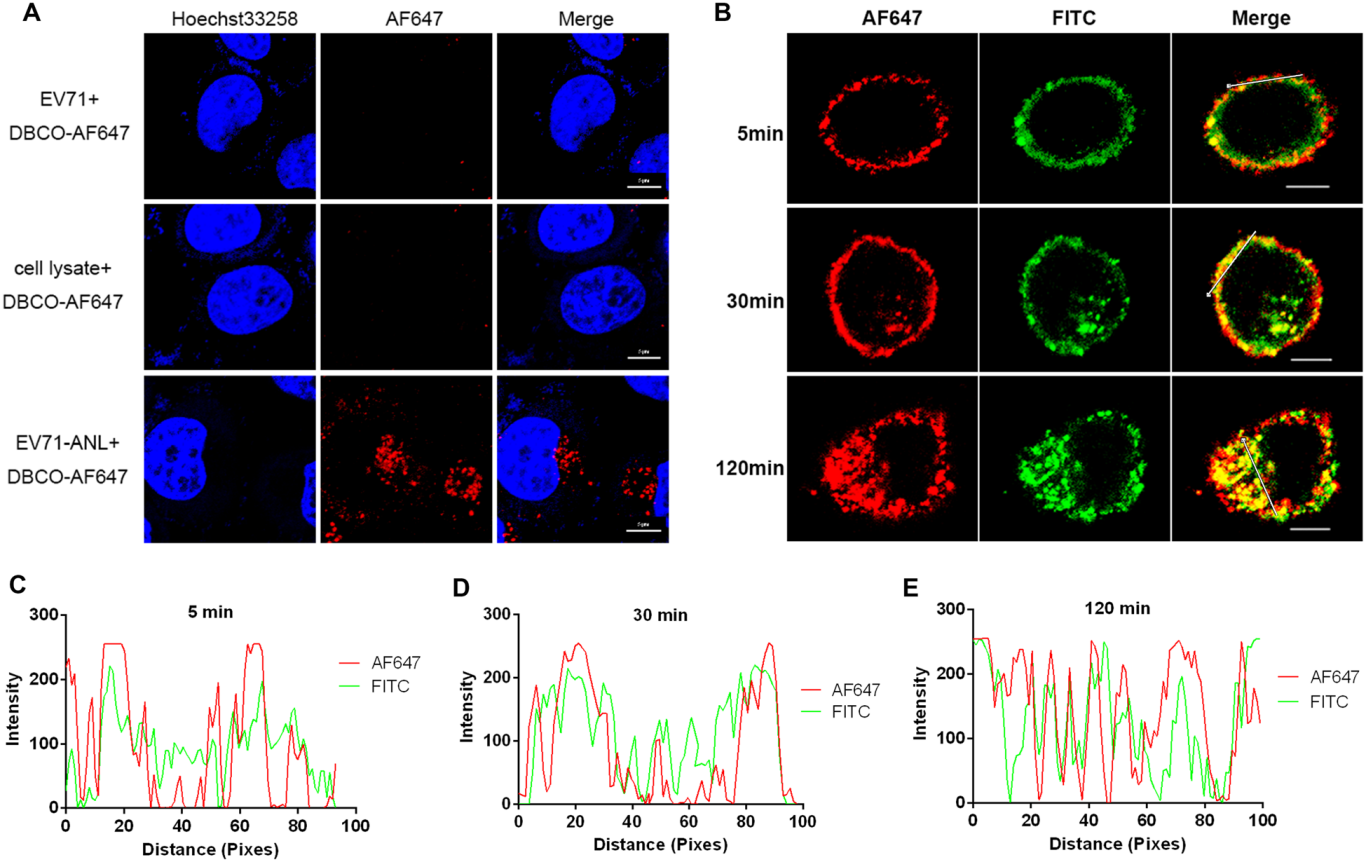
Characterization of AF647-labeled EV71. **A** EV71, EV71-ANL, or RD-MetRS*-ANL cell lysates were mixed with DBCO-AF647 and then used to infect HeLa cells for 2 h. Scale bar: 10 μm. **B** EV71-AF647-infected HeLa cells were fixed and incubated with anti-VP1 antibody and FITC-conjugated secondary antibody. Scale bar: 10 μm. **C–E** Line profile analysis of AF647 and VP1 signals of EV71-AF647-infected cells at 5, 30, and 120 min post-infection. Lines were shown in the merged channels in **B**

### EV71 virus can be efficiently labeled with ANL and DBCO-dye

We next determined whether EV71 could be successfully fluorescent-labeled with this system. EV71 virus was propagated in RD-MetRS* cells cultured with ANL and purified to obtain EV71-ANL for fluorescence labeling. EV71-ANL was stained by DBCO-AF647 and purified to obtain EV71-AF647 particles. To confirm whether EV71 could be specifically labeled through our system and whether EV71-AF647 particles could infect cells efficiently, HeLa cells were incubated with (a) the mixture of EV71 and DBCO-AF647, (b) the mixture of RD-MetRS*(RM)-ANL cell lysates and DBCO-AF647 and (c) EV71-AF647 particles respectively and then washed to remove the dyes which cannot be uptaken by cells. Compared with the other groups, EV71-AF647 particles exhibited a different fate. As shown in Fig. 3A, cells incubated with EV71-AF647 showed strong fluorescent signals. In contrast, no signal could be observed in cells incubated with the mixture of DBCO-AF647 dye and EV71. To exclude the possibility of contamination with ANL-labeled cellular proteins, lysates of noninfected RD-MetRS* cells cultured with ANL were subjected to the same purification, labeling, and infection procedures. However, the cells in the lysate group showed no AF647 signal.

The labeling specificity was also confirmed by immunofluorescence assay (Fig. 3B). After the EV71-AF647 adsorbing on the cell surface and infecting cells for different time points, an antibody against the EV71 and FITC-conjugated secondary antibody was added to detect viral particles. EV71-FITC signals showed high colocalization with that of DBCO-AF647, as suggested by the line profile analysis of AF647 and EV71-FITC signals on the cell surface (Fig. 3C). Moreover, both fluorescent signals moved from the cell surface toward the cell cytoplasm with increasing infection time, and the colocalization of FITC and AF647 signals at high efficiency was observed at all the indicated time points (Fig. 3C–E). Thus, AF647 signals could be used to monitor virus movement in infected cells.

**Fig. 4:**
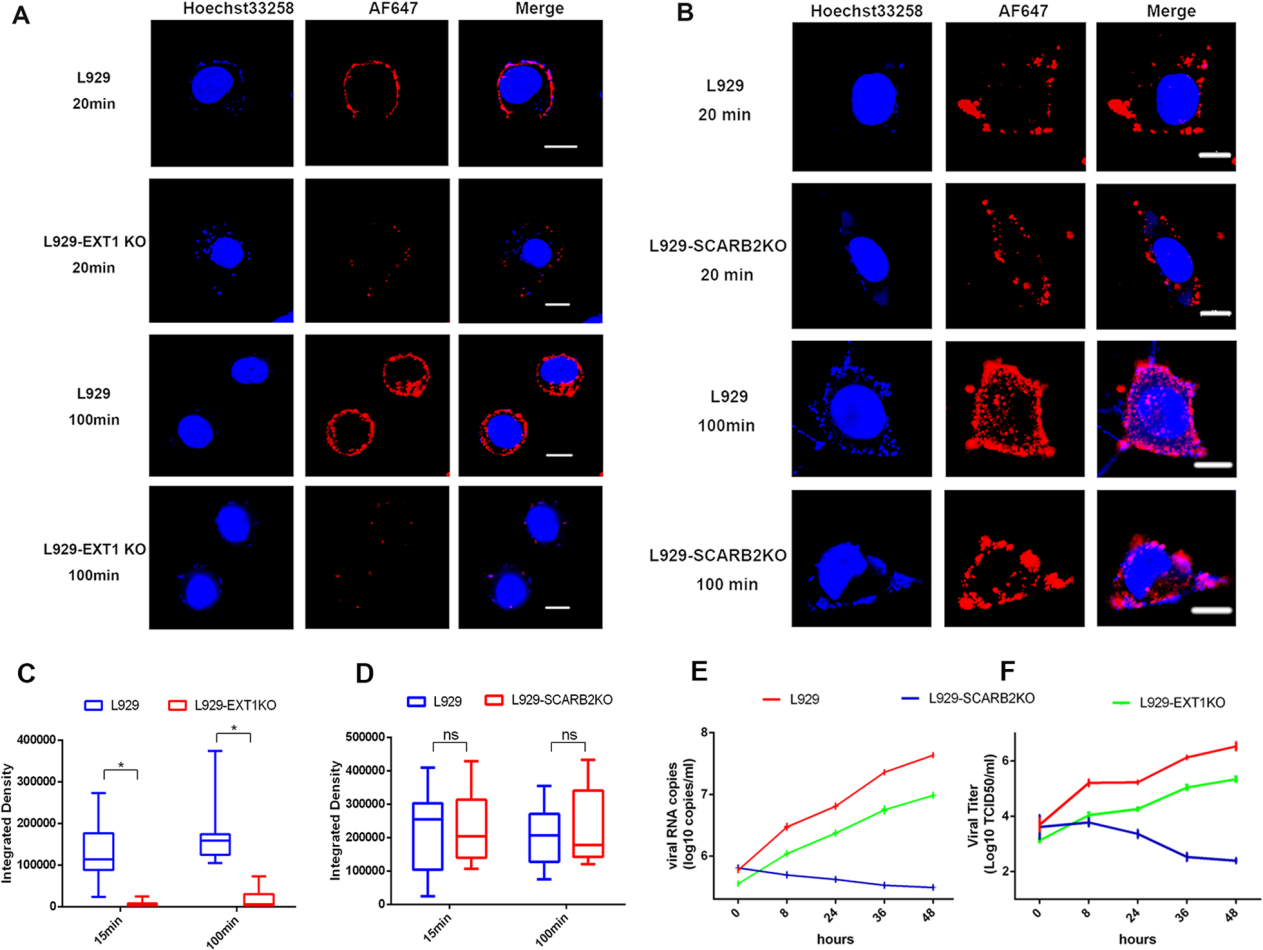
Infection and replication of EV71 in L929, L929-SCARB2KO, and L929-EXT1KO cells. **A**, **B** AF647 signals in different cells were detected at 20 or 100 min post-infection. Scale bar: 10 μm. **C**, **D** Densities of AF647 Fluorescent signals in ~ 20 L929, L929-SCARB2KO, and L929-EXT1KO cells at 20 or 100 min post-infection were quantified with the Fiji Image J software. *p < 0.05, ns: not significant. Viral RNA copy numbers (**E**) and Virus titers (**F**) were detected at the indicated time points. The time point when the incubation period (1 h) was over was set as timepoint zero

### Analysis of receptor usage by labeled EV71 virus

EV71 virus can use multiple cellular proteins or glycans as receptors [3]. Among them, HS and SCARB2 protein were found to play important roles in the initial infection stage. To compare the usage of the two receptors by EV71, EV71-AF647 dynamics were monitored in L929 cell lines in which HS or SCARB2 was deficient due to EXT1 (essential for HS biosynthesis) or SCARB2 knockout [27]. In HS-depleted L929-EXT1KO cells, virus attachment to the cell surface was significantly reduced (Fig. 4A). Little EV71-AF647 signal was observed at either 20 or 100 min poi. In contrast, a strong AF647 fluorescent signal accumulated at L929 cell surface at 20 min poi, and some signals had shifted to the cytoplasm at 100 min poi (Fig. 4A). SCARB2 knockout showed only a moderate effect on EV71 attachment (Fig. 4B). The results were also supported by quantitative analysis of the AF647 signals in L929, L929-EXT1KO or L929-SCARB2KO cells (Fig. 4C-D). Interestingly, As shown in Fig. 4E and F, after entering cells, the viral RNA copy number and virus titer in L929-SCARB2KO cells decreased as the infection time increased, revealing a SCARB2-dependent replication mechanism of EV71. In contrast, although HS depletion showed a strong effect on virus attachment, the viral RNA copy number or virus titer showed growth kinetics similar to those in L929-EXT1KO cells but decreased by nearly 100-fold. Overall, the results suggested that although SCARB2 was crucial for virus infection, it was nonessential for cell attachment. It may play an essential role in EV71 uncoating or replication rather than binding and early entry stage. On the other hand, HS plays a more important role in cell binding but cannot enable successful infection and replication without SCARB2.

**Fig. 5:**
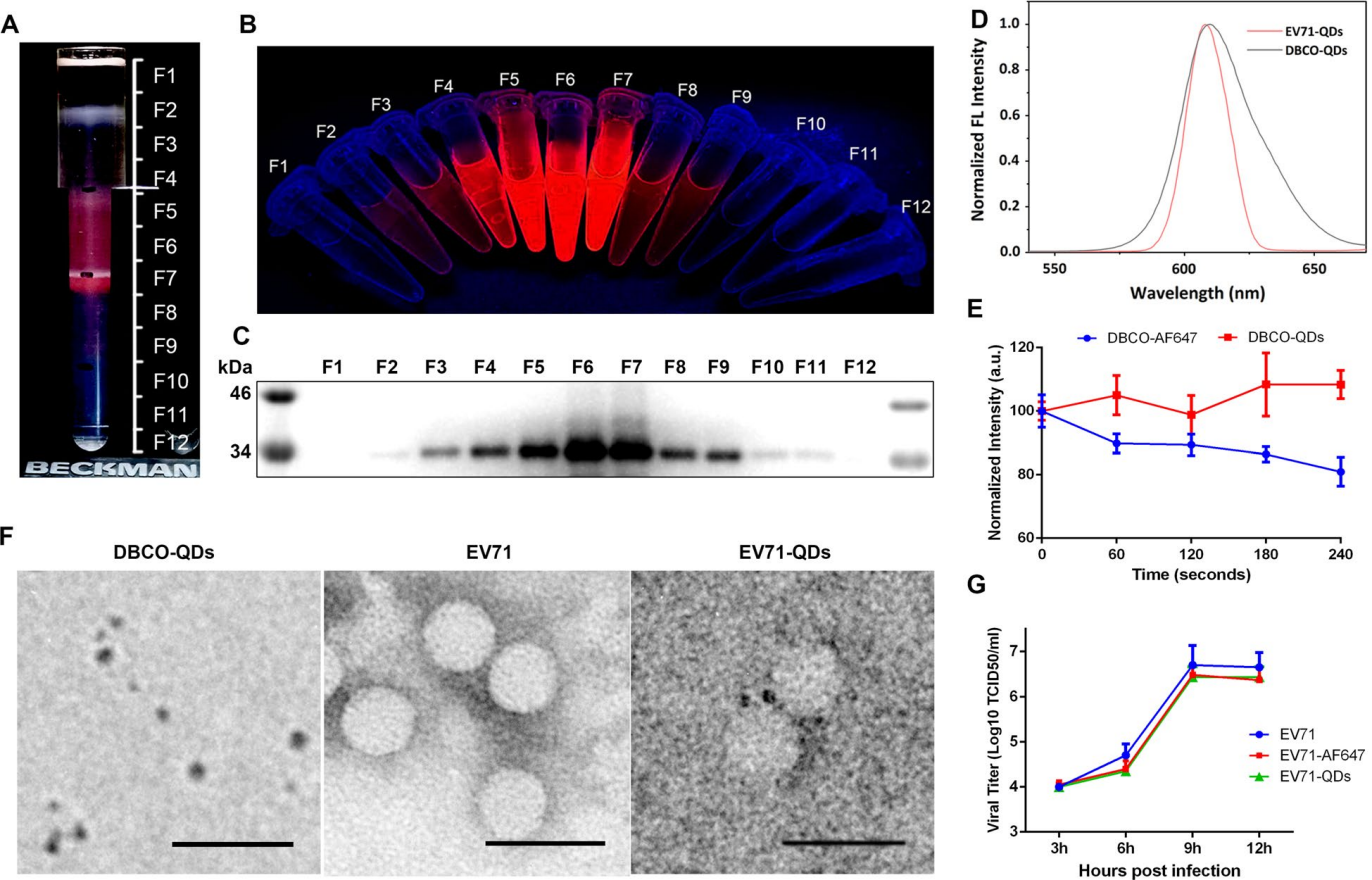
Purification and characterization of EV71-QDs. **A** Image of the DGC tube after the separation of EV71-QD under bright light. **B** Image of fractions F1 ~ F12 under a UV lamp. **C** VP1 proteins in F1 ~ F12 were analyzed by Western blotting to confirm the presence of EV71. **D** Fluorescence spectra of DBCO-QDs and EV71-QDs. **E** DBCO-QDs (8 nM) and DBCO-QF647 (2.5 µM) were continuously illuminated by a laser at 488 nm or 633 nm and kinetics of the fluorescence intensity were determined by a microplate reader in four minutes. **F** TEM images of DBCO-QDs, EV71, and EV71-QDs. Scale bar: 50 nm. **G** One-step growth curve of EV71, EV71-AF647, and EV71-QDs

### Characterization of QD-labeled EV71

Chemical dyes usually undergo rapid photobleaching and are not suitable for dynamic virus tracking. QD is an alternative material that can be suitable for long-term real-time imaging owing to its remarkable brightness and photostability. The most commonly used strategy for labeling viruses with QDs is based on the reaction between streptavidin conjugated to QDs (SA-QDs) and biotin bound to the target virus. However, the size of QDs-SA can be up to 24nm because of the large size of streptavidin. SA-QDs are not suitable for small-sized viruses such as EV71 which has a diameter of less than 30 nm. Modifying QDs with small chemical groups through click chemistry ligation would not notably increase the size of QDs. Fortunately, the size of QDs can even decrease by 15 % compared to QDs when conjugated with DBCO due to the hydrophobicity of DBCO [29]. Therefore, we attempted to label EV71-ANL with DBCO-QDs through click chemistry (Fig. 5). EV71-ANL was mixed with DBCO-QDs for reaction and then separated by density gradient centrifugation (DGC). DGC sample aliquots containing both QD signals under UV (Fig. 5A, B) and VP1 proteins (Fig. 5C) were observed under TEM to detect EV71-QD conjugates. Virus particles conjugated with QDs can be observed in Fig. 5F. Fluorescence spectra analysis revealed an ~ 5 nm blueshift in the DBCO-QDs wavelength, which may be caused by viral proteins on QDs. To determine whether the labeling procedure impairs EV71 infectivity, a growth curve assay was performed in RD cells (Fig. 5G). Compared with wild-type EV71 virus, both AF647- and QD-labeled viruses replicated at lower titers at different time points (6 h, 8 h, and 12 h) post-infection, but the differences were not statistically significant, indicating that the labeling method did not reduce viral infectivity. Because AF647 underwent photobleaching rapidly after continuous excitation (Fig. 5E), EV71-QDs were used for dynamic visualization studies.

**Fig. 6:**
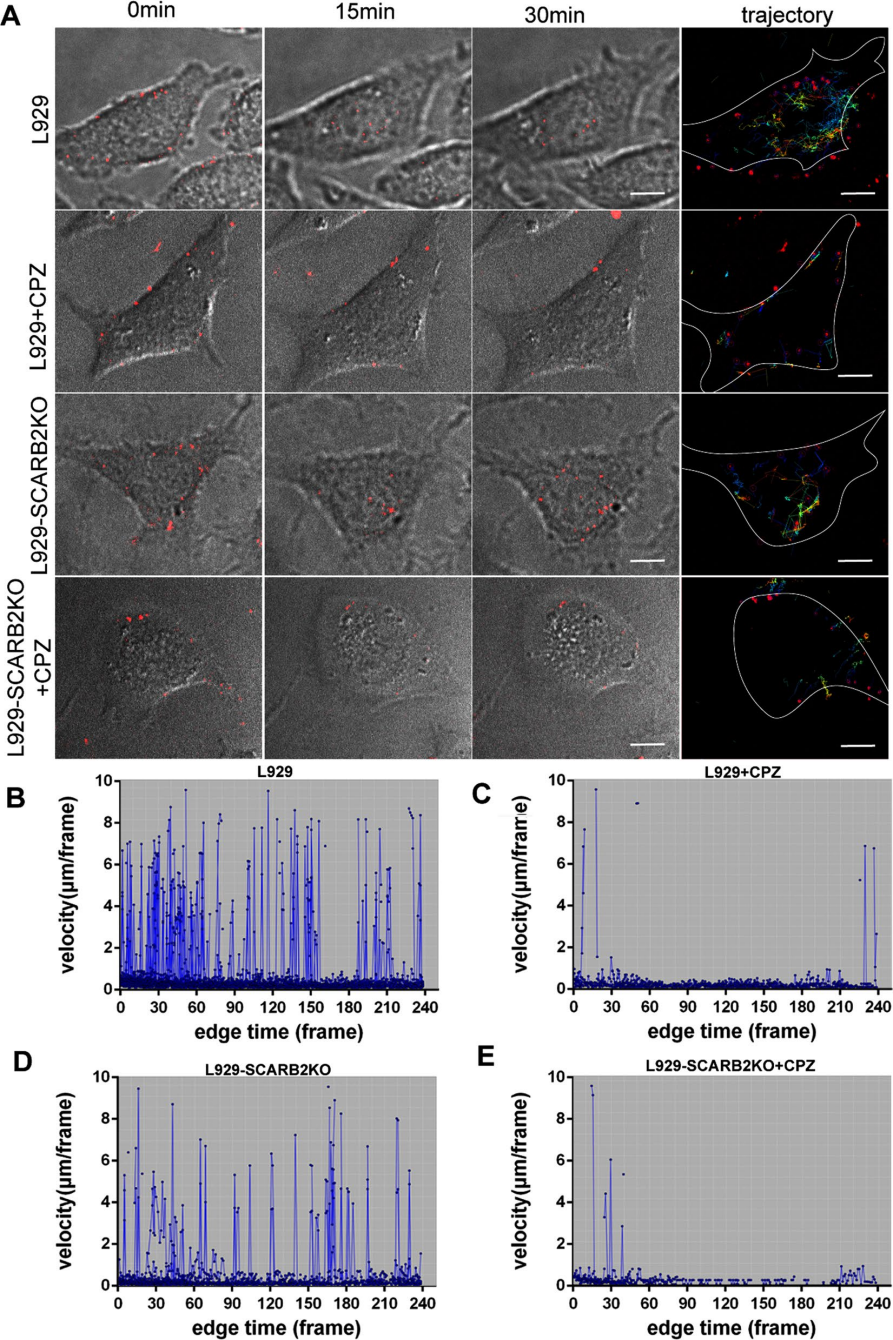
Dynamics of EV71-QD movement in L929 or L929-SCARB2KO cells. L929 or L929-SCARB2KO cells infected with EV71-QDs were untreated or treated with chlorpromazine (CPZ), and the locations and trajectories (color lines) of QD signals (red dots) in cells are shown in **A**. The average velocity of EV71-QDs in CPZ-treated (**C**, **E**) or untreated (**B**, **D**) cells in 240 continuous frames (in 30 min) was analyzed. Scale bar: 10 μm

### Dynamic visualization of SCARB2-independent entry of EV71 using EV71-QDs

Since EV71 could enter cells without SCARB2, we analyzed this entry stage through dynamic visualization using the EV71-QDs (Fig. 6). L929 or L929-SCARB2KO cells were incubated with EV71-QDs for attachment and then subjected to live cell imaging (Fig. 6). Bright QD signals were detected at the cell membrane in both cell lines at time point 0. As time passed, the QD signals gradually moved from the periphery toward the cytoplasm. At 15 and 30 min post-infection, EV71-QDs appeared mainly in the cytoplasm of L929 cells, although some fluorescent signals could still be observed at the cell surface (Fig. 6A, B). When we treated L929 cells with chlorpromazine (CPZ), an inhibitor of the clathrin-mediated endocytosis (CME) process, the entry of EV71 was blocked (Fig. 6A, C). This result is consistent with the CME entry pathway of EV71 reported previously [30]. To track the early stage of EV71 entry L929-SCARB2KO cells, we also treated the L929-SCARB2KO cells with or without CPZ before and after EV71 infection. As indicated in Fig. 6, EV71-QD signals were trapped at the cell membrane in CPZ-treated cells, but uptaken efficiently by untreated cells, regardless of whether SCARB2 was knocked out. Trajectory analysis revealed that the EV71-QDs moved toward the cell center in L929 and L929-SCARB2KO cells, which indicated virus internalization. However, in CPZ-treated cells, EV71-QDs moved in a limited area near the cell. Velocity analysis also showed a lower speed of EV71-QDs in CPZ-treated cells. Taken together, these results revealed the CME pathway of EV71 in SCARB2KO cells. It confirmed that as a receptor of EV71, SCARB2 is dispensable in viral attachment and early entry stage, which was not previously reported.

## Conclusion

In this study, we developed a universal bioorthogonal labeling system for labeling viruses with dyes and QDs and real-time tracking of the early virus infection steps. Unlike the FP-fusion method which may prevent virus assembly, or chemical labeling which usually disrupts the charged residues essential for virus-cell interaction, our system doesn’t interfere with the replication or impair the infectivity during labeling. Besides, compared with other UAA-labeling methods, our system does not require a methionine-free environment owing to the high ANL incorporation efficiency of the MetRS* cell line, thus shows no effect on cell viability and virus yield. The EV71-ANL particles can be easily labeled with DBCO-probes to track virus movements in living cells. When labeled with DBCO-QDs, the long-time real-time tracking of EV71 can be performed. In addition, this method does not require the construction of recombinant viruses for labeling through reverse genetic methods. Instead, it requires only a permissive cell line that expresses MetRS*, which can be easily constructed. Except for RD-MetRS* cells, we also constructed MetRS* stably expression Vero cell line (data not shown), which can be used for propagation and labeling of many important human viruses, such as coronavirus, flavivirus, and picornavirus.

Using the labeled EV71 GZCII-P30 strain and two receptor depletion cells, we found that cellular HS is dispensable for virus replication but can significantly induce virus binding to the cell surface. On the other hand, SCARB2, another important EV71 receptor, has a limited function in cell attachment but is essential for EV71 infection and replication. Our study provides a dynamic infection process and direct evidence of the distinct functions of HS and SCARB2 in EV71 infection. Importantly, EV71 can enter SCARB2-knockout cells efficiently, probably through other receptors. And the internalization can be inhibited by chlorpromazine. We used the EV71-GZCII-P30 virus, a strong HS-binding strain, in this study. The entry mechanisms of other poor HS-binding EV71 strains may also be studied using the same method. Moreover, our findings regarding EV71 receptor utilization may also help to explain their roles in EV71 pathogenesis.

In a word, our study provides a convenient method for labeling viruses with high efficiency and robust virus yield. In principle, the method could be applied to many other viruses. Also, it takes advantage of the excellent fluorescence property of QDs and may represent a powerful tool for elucidating the molecular details of entry and intracellular transport of many kinds of viruses to study their receptors and infection mechanism.

## Supplementary Material

**Additional file 1: Table S1.** Primers used for molecular cloning.** Figure S1.** The titer of EV71-GZCII produced by ANL-based system and AHA-based system detected by crystal violet staining assay.

## Data Availability

The datasets and materials used in the study are available from the corresponding author.
