# Supplementary material for: Metabolic labeling of enterovirus 71 with quantum dots for the study of virus receptor usage
Source: J Nanobiotechnology. 2021 Sep 28;19:295. doi: 10.1186/s12951-021-01046-5 (PMC8477995; doi:10.1186/s12951-021-01046-5)
Supplement: Supplementary file 1 — Additional file 1: Table S1. Primers used for molecular cloning. Figure S1. The titer of EV71-GZCII produced by ANL-based system and AHA-based system detected by crystal violet staining assay. [file 12951_2021_1046_MOESM1_ESM.docx]

**Table S1. Primers used for molecular cloning**

| **primers** | **Sequences (5’ to 3’)** | **description** |
| --- | --- | --- |
| MetRS-F | atggattacaaggatgacgacgataagatgagactgttcgtgagtgatggcg | for amplifying MetRS gene |
| MetRS-R | ttactttttcttcttgcctttaggggcttcagg |  |
| MetRS*-F(EcoRI) | ccaccgaattcgccaccatggattacaaggatgacgacgataagatga | for restriction cloning (restriction site underlined) |
| MetRS*-R(NotI) | ccaccgcggccgcttactttttcttcttgcctttaggggcttca |  |
| MetRS*-2F | gaaaggaatgtgctcatcaccagtgccGGAccttacgtcaacaatgtcccccac | for introducing L272G mutation （mutation sites capitalized） |
| MetRS*-2R | gtgggggacattgttgacgtaaggTCCggcactggtgatgagcacattcctttc |  |

**
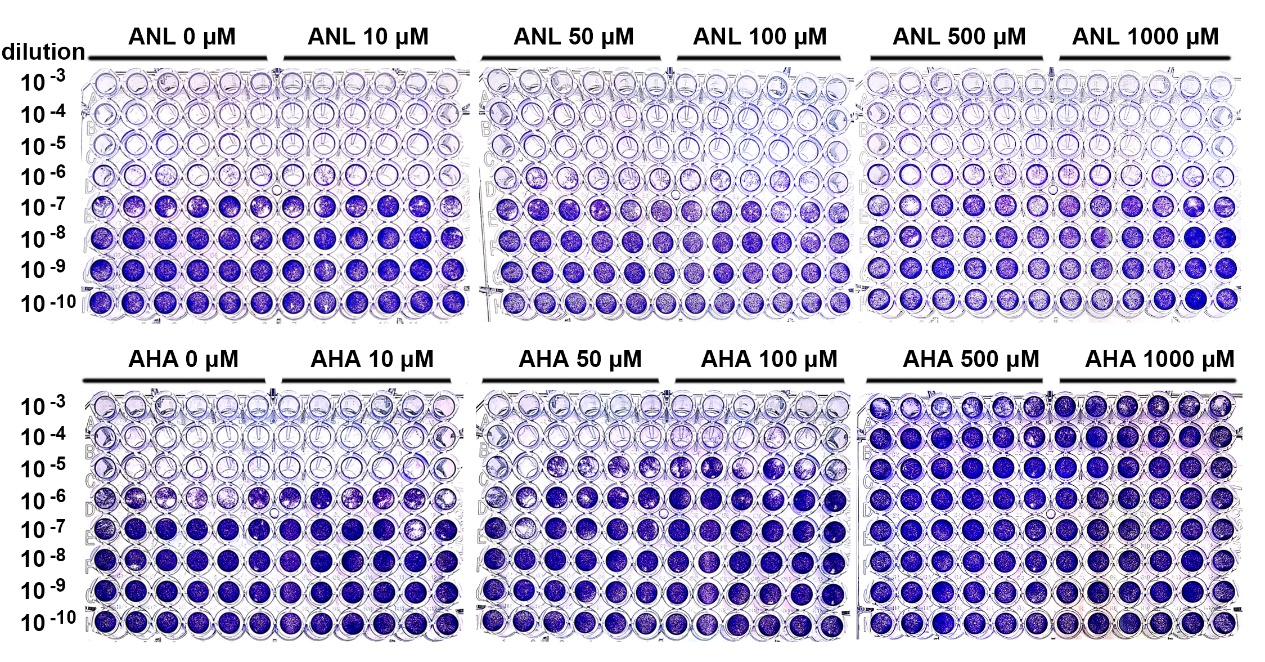
Figure S1. The titer of EV71-GZCII produced by ANL-based system and AHA-based system detected by crystal violet staining assay.**
